# Atovaquone-proguanil in the treatment of imported uncomplicated *Plasmodium falciparum* malaria: a prospective observational study of 553 cases

**DOI:** 10.1186/1475-2875-12-399

**Published:** 2013-11-07

**Authors:** Hugues Cordel, Johann Cailhol, Sophie Matheron, Martine Bloch, Nadine Godineau, Paul-Henri Consigny, Hélène Gros, Pauline Campa, Patrice Bourée, Olivier Fain, Pascal Ralaimazava, Olivier Bouchaud

**Affiliations:** 1 Department of Infectious and Tropical Diseases, Hôpital Avicenne, Assistance Publique-Hôpitaux de Paris, Bobigny, France; 2 LEPS EA3412, Université Paris 13 - Sorbonne Paris Cité, Paris, France; 3 Department of Infectious and Tropical Diseases, Hôpital Bichat, Assistance Publique-Hôpitaux de Paris, Paris, France; 4 Department of Internal Medicine, Hôpital Louis Mourrier, Assistance Publique - Hôpitaux de Paris, Colombes, France; 5 Department of Parasitology, Hôpital Delafontaine, Saint Denis, France; 6 Institut Pasteur, Centre Médical, Paris, France; 7 Department of Internal Medicine, Hôpital Robert Ballanger, Aulnay-Sous-Bois, France; 8 Department of Infectious and Tropical Diseases, Hôpital Saint Antoine, Assistance Publique - Hôpitaux de Paris, Paris, France; 9 Department of Parasitology, Hôpital Bicètre, Assistance Publique - Hôpitaux de Paris, Paris, France; 10 Department of Internal Medicine, Hôpital Jean verdier, Assistance Publique - Hôpitaux de Paris, Paris, France

**Keywords:** Atovaquone-proguanil, Imported, Uncomplicated, Malaria, Tolerance

## Abstract

**Background:**

Each year, thousands of cases of uncomplicated malaria are imported into Europe by travellers. Atovaquone-proguanil (AP) has been one of the first-line regimens used in France for uncomplicated malaria for almost ten years. While AP’s efficacy and tolerance were evaluated in several trials, its use in “real life” conditions has never been described. This study aimed to describe outcome and tolerance after AP treatment in a large cohort of travellers returning from endemic areas.

**Methods:**

Between September 2002 and January 2007, uncomplicated malaria treated in nine French travel clinics with AP were followed for 30 days after AP initiation. Clinical and biological data were collected at admission and during the follow-up.

**Results:**

A total of 553 patients were included. Eighty-eight percent of them were born in Africa, and 61.8% were infected in West Africa, whereas 0.5% were infected in Asia. Migrants visiting friends and relatives (VFR) constituted 77.9% of the patients, the remainder (32.1%) were backpackers. Three-hundred and sixty-four patients (66%) fulfilled follow-up at day 7 and 265 (48%) completed the study at day 30. Three patients had treatment failure. One-hundred and seventy-seven adverse drug reactions (ADR) were reported during the follow-up; 115 (77%) of them were digestive ADR. Backpackers were more likely to experiment digestive ADR compared to VFR (OR = 3.8; CI 95% [1.8-8.2]). Twenty patients had to be switched to another regimen due to ADR.

**Conclusion:**

This study seems to be the largest in terms of number of imported uncomplicated malaria cases treated by AP. The high rate of reported digestive ADR is striking and should be taken into account in the follow-up of patients since it could affect their adherence to the treatment. Beside AP, artemisinin combination therapy (ACT) is now recommended as first-line regimen. A comparison of AP and ACT, in terms of efficacy and tolerance, would be useful.

## Background

In 2010, the World Health Organization (WHO) estimated that 216 million cases of malaria occurred and were responsible of 650,000 deaths [1]. More and more people travel from northern countries to malaria-endemic areas. As a result, imported malaria is not exceptional in Europe, where approximately 7,000 cases were notified in 2010 to the WHO Regional Office for Europe [2]. The actual case numbers might be higher, due to under-notification. With its historical links with West and Central Africa, France is the leading European country in terms of cases of malaria diagnosed with 3,560 cases reported in 2011 by the National Malaria Reference Centre [3]. In Europe and other northern countries, travellers are treated by highly effective anti-malarial therapy, by contrast to malaria-endemic countries where appropriate treatments are not always available. A recent publication has shown that 18 different regimens were used in Europe, with the most prescribed being the fixed combination, atovaquone-proguanil (AP), commercially known as Malarone® [4].

AP has been licensed in France since 1997 and marketed since late 2001 and it remains the principal treatment of acute malaria. Since the National Experts Committee recommended in 2007 that AP, together with artemether-lumefantrine, be used as first-line regimen, ‘old drugs’ such as quinine or mefloquine were downgraded to second-line treatment [5]. Atovaquone belongs to the family of hydroxy-naphthoquinones, which anti-malarial activity was first described 40 years ago [6,7]. Its mode of action is original, blocking the electron transport chain of the parasite’s mitochondria [8]. Used on its own, atovaquone has limited value, as shown by a significant relapse rate [9]. Its association with proguanil has shown excellent efficacy on acute malaria in numerous clinical trials, due to a synergistic effect [10-17]. AP is also widely used as an efficient and well-tolerated chemoprophylaxis for travellers.

Efficacy and tolerance of AP were extensively reported from clinical trials performed in malaria-endemic areas [10-14]. However, these results might not be valid in the case of imported malaria, due to epidemiological and biological differences (i e, study conditions, immune status, parasitaemia, heterogeneity of parasite strains, perception of side effects). Most of the studies comparing AP to other drugs were performed in endemic countries, and few observational or retrospective studies from non endemic countries have been published, amongst which only was a comparative trial, yet with a limited number of subjects has been published [18-24]. A recent international, prospective, observational study analysed a large cohort (504 cases) of imported *Plasmodium falciparum* malaria cases. It provided interesting data on the use of AP with a relatively large series (n = 253), but heterogeneity of practices, due to ‘centre effects’ between participating European centres may limit the interpretation of the study [4].

The aim of this study was to bridge this gap in knowledge by providing data on the use of AP in ‘real life’ conditions in France, using a large series of uncomplicated imported cases of *P. falciparum*.

## Methods

### Study design

A multicentre, prospective, observational study was set up in nine travel clinics located in Paris and its suburbs, between September 2002 and January 2007. Written informed consent was obtained from each participating subject (from accompanying parents for children less than 18 years old) and data management procedures were approved by the French Commission National de l’Informatique et des Libertés (CNIL).

### Study population

Recruitment criteria were as following: more than 12 years old (minimal age for prescribing the standard dosage of AP according to manufacturer recommendations); an acute malaria onset due to *P. falciparum,* acquired in an endemic country and imported to France; onset treated with AP; and, having signed an informed consent. Mixed infections, defined by an infection to *P. falciparum* combined with another species of *Plasmodium* were also included. Diagnosis of malaria was based on positive Giemsa-stained thin and thick blood smears tests performed by the parasitology laboratory in each participating centre. All patients had to tolerate oral therapy when they were included in the study. Patients were excluded if: they fulfilled any of the clinical and biological WHO criteria of severe or complicated malaria [25], particularly parasitaemia higher than 4% even for immune patients; if they had a history of allergy to AP; if pregnant or breast-feeding. Hospitalization of patients was not required if the clinical status was sufficiently good and if compliance to the treatment and follow-up was foreseen as acceptable. Patients initially treated with less than three days of intravenous quinine because of vomiting at admission were not excluded as tolerance data in that particular situation were of interest. Decision for choosing AP or another anti-malarial drug was the responsibility of each investigator. Data were collected via a standardized datasheet and analysed only for patients treated with AP.

### Procedures/data collection

Initial evaluation included individual characteristics (date of birth, sex, country of birth, country of residence), details on travels in malaria-endemic areas (date of arrival in France, countries visited and duration of travels), type of malaria prophylaxis, medical history (co-morbidities and previous malaria onsets) and details on actual onset. A semi-immune person was defined as a patient who declared a history of malaria. Clinical examination was followed by: blood smear; laboratory test exploring liver, kidney, haematological and metabolism functions; and, an electrocardiogram. According to international and French follow-up guidelines, patients were monitored at day 3, day 7 and one month (day 28 to 30) after AP initiation with clinical examination, blood smear and haematological, liver and kidney functions. Doses for AP and all anti-malarial drugs, except mefloquine, is one tablet daily for chemoprophylaxis. Compliance to daily chemoprophylaxis during the travel period was evaluated during the initial interview: a good compliance was defined by one or less missed dose by week during the travel period and one month after return. For mefloquine (MQ), compliance was defined by one or less missed dose during the travel period and one month after return. Appropriate use of exposure prophylaxis was defined as patients regularly using impregnated bed net and skin repellent.

Apyrexia (fever clearance) was defined as a tympanic temperature below 37.8°C and was monitored at day 3, day 7 and one month. Parasite clearance was defined as a negative thin/thick blood film and was monitored at day 3, day 7 and one month.

Adverse drug reactions (ADR) were reported using a questionnaire administered by the physician. An assessment of patients’ perception of tolerance was performed, using the following terms: good, satisfactory, bad, and very bad tolerance. In the same way, assessment of patients’ perception of AP efficacy was performed. Patients were asked their subjective feeling about the treatment, using the following items: efficient, moderately, poorly and not efficient in the questionnaire, without clinical or biological data. Data were captured on a standardized datasheet and transmitted to the study principal investigator at the end of follow-up. A limited number of patients were also included in another observational study (the European Malther study) recently published [4].

### Data analysis

Statistical analysis was performed using Stata® version 10 software (StataCorp LP, 4905 Lakeway Drive, College Station, TX 77845, USA). Descriptive analyses were comprised of frequency distributions and proportions for each variable category, with their quartiles and confidence intervals (CI) 95%. Group comparisons were performed using Chi square test and Fisher’s exact test for categorical variables. Spearman's rank correlation coefficient was used to assess association between two continuous variables. Logistic regression analysis was performed to measure association between digestive ADR and patient’s characteristics as independent variables: sex, age group, country of birth, chronic illness (cardiopathy, obesity, diabetes mellitus, kidney and respiratory diseases, HIV infection), type of chemoprophylaxis, type of travel, parasitaemia at diagnosis and at day 3 nausea at presentation, defined as nausea reported at diagnosis, and immunity, classified in semi-immune person or non immune”.

Odds ratios (OR) and 95% CI were calculated from β coefficients and their standard errors. Variables with a *p-*value < 0.30 were included in the adjusted model.

## Results

### Population

During the study period, 553 patients met eligibility criteria and were enrolled in the study. The median age of patients was 38.3 years old (12–79) and 66% were male (sex ratio 1.9). The majority of patients (90.8%) were born outside Europe, mainly in Africa (88.6%). Information on co-morbidities was available for 478 (86.4%): 21 were HIV positive (4%); and 44 (7.9%) had a cardiopathy. Nearly half of enrolled patients (n = 264; 47.7%) declared at least one previous onset of malaria.

Countries of contamination were mainly located in Africa, especially West Africa for 342 (61.8%) travellers. Only three (0.5%) and two (0.4%) were infected in Asia and the Caribbean Islands, respectively. Most of the patients were migrants who visited friends and relatives (VFR) (n = 431, 77.9%). Others were backpackers (n = 33, 6.0%) and tourists resident in hostels (n = 11, 2.2%).

A pre-travel consultation was reported in 267 travellers (48.3%), mostly by their family practitioner (42.3%). Two-hundred and twenty-two patients (40.1%) declared having taken malaria chemoprophylaxis (chloroquine-proguanil in 64% of the cases). Information on compliance to chemoprophylaxis was available for 222 subjects and was classified as good for 50 cases (22.5%). Seventy-eight subjects (14.1%) declared having used exposure prophylaxis, including 12 (2.2%) with appropriate exposure prophylaxis, i e, regular use of impregnated bed net and skin repellent. Twenty patients (3.6%) experienced digestive disorders during their stay. There was no relationship between malaria chemoprophylaxis and digestive disorders (*p* = 0.14). Twenty-two patients were treated by intravenous quinine (14 patients for 24 hours or less, four for 48 hours and four for 72 hours) before receiving AP because of vomiting at admission. Table 1 summarizes the main characteristics of patients enrolled.

**Table 1:** Main characteristics of 553 patients treated with atovaquone-proguanil for imported uncomplicated malaria

	
**Male: female ratio**	1.9
**Median age in years**	38.3 (12–79)
**Median weight (kg)**	74 (40–109)
**Continent of birth N (%)**	
Europe	51 (9.2)
French West Indies	4 (0.7)
Africa	490 (88.6)
Others	8 (1.5)
**Previous history of malaria**	264/492* (53.7)
**HIV positive**	21/478* (4.4)
**Pre-travel visit**	267 (48.3)
Travel clinic	74 (27.7)
Family doctor	113 (42.3)
Unknown	80 (30.0)
**Chemoprophylaxis**	222 (40.1)
Chloroquine	30 (13.5)
Chloroquine-proguanil	142 (64)
Mefloquine	24 (10.8)
Atovaquone-proguanil	1 (0.4)
Doxycycline	13 (5.9)
Proguanil	10 (4.5)
Unknown	2 (0.9)
**Compliance to chemoprophylaxis**	50/222* (22.5)
**Non-medical prophylaxis**	
Air-conditionning only	1 (0.2)
Insecticides only	5 (0.9)
Unimpregnated bed net only	11 (2)
Impregnated bed net only	23 (4.1)
Repellents only	23 (4.1)
Impregnated bed net and repellents	12 (2.2)
No prophylaxis	400 (72.4)
Unknown	78 (14.1)
**Place of contamination**	
West Africa	342 (61.8)
Central Africa	148 (26.8)
Madagascar and Comoros	51 (9.2)
East Africa	5 (0.9)
Asia	3 (0.5)
Angola and South Africa	2 (0.4)
Haïti and French Guyana	2 (0.4)
**Type of travel**	
Backpackers	33 (6.0)
Hostel	11 (2.0)
Visiting friends and relatives	431 (77.9)
Unknown	78 (14.1)

*: number of patients with available data.

### Diagnosis

The median time between onset and diagnosis was five days [1–64]. Initial clinical presentation comprised headache (46.3%), nausea and vomiting (28.2%), diarrhoea (18.5%), myalgia (14.7%), abdominal pain (5.8%) and arthralgia (3.6%). For 175 patients (31.6%), no fever was noted at admission (Table 2).

**Table 2:** Admission variables and outcome in 553 patients treated with atovaquone-proguanil for imported uncomplicated malaria

**Temperature at diagnosis N (%)**	
≤37.7C°	175 (31.6)
37.8-39C°	208 (37.6)
39.1-40C°	104 (18.8)
>40C°	19 (3.4)
**Symptoms at diagnosis**	
Headache	256 (46.3)
Nausea/vomiting	156 (28.2)
Diarrhoea	102 (18.5)
Myalgia	81 (14.7)
Abdominal pain	32 (5.8)
Arthralgia	20 (3.6)
**Compliance to follow-up**	
Day 3	469 (84.1)
Day 7	364 (65.8)
Day 30	265 (47.9)
**Hospitalization**	
Hospitalized at diagnosis	191 (34.5)
Still hospitalized at day 3	124 (22.4)
Still hospitalized at day 7	15 (2.7)
Still hospitalized at day 30	2 (0.3)
**Fever clearance (apyrexia)**	
Day 3	403/425* (94.8)
Day 7	323/323* (100)
Day 30	227/227* (100)
**Parasitological clearance (negative parasitaemia)**	
Day 3	292/425* (68.7)
Day 7	331/331* (100)
Day 30	215/217* (99.1)

*: number of patients with available data.

### Biology

The main data are summarized in Table 3. All patients were infected by *P. falciparum*. In two cases *P. falciparum* was associated with another species: one with *Plasmodium vivax* from India and one with *Plasmodium ovale* from Gabon.

**Table 3:** Biology data in 553 patients treated with atovaquone-proguanil for imported uncomplicated malaria

	**Diagnosis**	**Day 3**	**Day 7**	**Day 30**
**Haemoglobin (g/dL)**	N (%)	N (%)	N (%)	N (%)
≤8	11 (1.9)	16 (3.4)	4 (1.1)	0
8.1-10	28 (5.1)	55 (11,7)	48 (13.2)	17 (6.4)
10.1-12	136 (24.6)	162 (34.6)	126 (34.6)	73 (27.6)
>12	378 (68.4)	236 (50.3)	186 (51,1)	175 (66.0)
**Platelets count (per mm**^**3**^**)**				
≤20,000	8 (1.4)	4 (0.8)	0	0
20,001-50,000	43 (7.8)	14 (3.0)	1 (0.3)	0
50,001-100,000	173(31.3)	102 (21.8)	1 (0.3)	0
100,001-150,000	160 (28.9)	121 (25.8)	6 (1.6)	7 (2,6)
>150,000	169 (30.6)	228 (48.6)	356 (97.8)	258 (97,4)
**Leucocytosis**				
White blood cells ≤4,500 per mm^3^	218 (39.4)	242 (51.6)	55 (15.1)	48 (18.1)
White blood cells >4,500 per mm^3^	335 (60.6)	227 (48.4)	309 (84.9)	217 (81.9)
**Cytolysis**				
ALAT ≤1 N	385 (69.6)	287 (61.2)	201 (55.2)	219 (82.6)
ALAT 1.1-2 N	135 (24.4)	140 (29.9)	117 (32.1)	44 (16.6)
ALAT 2.1-3 N	27 (4.9)	24 (5.1)	20 (5.5)	2 (0.8)
ALAT >3 N	6 (1.1)	18 (3.8)	26 (7.1)	0
**Creatinine (μmol/L)**				
≤120	507 (91.7)	431 (91.9)	349 (95.9)	4 (1.5)
121-140	36 (6.5)	25 (5.3)	8 (2.2)	253 (95.5)
>140	10 (1.8)	13 (2.8)	7 (1.9)	8 (3.0)

Median parasitaemia at diagnosis was 0.52% of red blood cell (0.01-5.0). Haemoglobin level was under or equal 8 g/dL at day 3 for 18 patients (3.9%) compared to ten (1.9%) at diagnosis (*p* < 10^-3^). There was no correlation between parasitaemia and haemoglobin level at diagnosis (*r* = −0.0017, *p* = 0.97). There was no correlation between the time elapsed between symptoms onset and diagnosis on one hand and haemoglobin level at diagnosis or at day 3 on the other hand (*r* = −0.21, p < 10^-3^ and *r* = −0.22, p < 10^-3^, respectively). At admission, platelets count was normal (>150,000/mm^3^) in 30.6% of cases and less than 20,000/mm^3^ in eight patients (1.5%). At day 7, 99.4% of patients had more than 100,000 platelets/mm^3^.

### Outcome

Follow-up was the following: 469 patients (85%) at day 3, 364 at day 7 (66%) and 265 patients (48%) completed the study one month after diagnosis. About one third of patients were hospitalized (n = 191, 34.5%). At day 3, 124 (22.4%) were still hospitalized and 15 (2.7%) at day 7. At one month, two patients were hospitalized: the first one was re-hospitalized for a relapse; the second one definitely cleared his parasitaemia at day 3 but was still hospitalized at day 30 for an HIV-related complication.

Fever clearance was obtained at day 3 in 95% of cases (403 of 425 for which information were available) and for all patients at day 7 (Table 2). All patients at one month were apyretic. Negative parasitaemia was observed in 68.7% of patients at day 3 and for all patients at day 7. Treatment failure was observed in three patients: two relapsed at day 30 and one at day 23. During follow up, these three patients did not return to an endemic area. All were successfully treated by mefloquine. Perceived efficacy amongst the 419 patients who answered was classified as efficient for 305 (72.8%), moderately for 101 (24.1%), poor for eight (1.9%) and not efficient for five (1.2%).

### Adverse drug reactions and drug switch

A total of 177 adverse drug reactions (ADR) attributed to AP were reported by the patients during the follow-up. Most of them were reported at day 3 (n = 150) and were digestive (n = 115, 77% of all ADR reported at day 3): most of them consisted of nausea and vomiting followed by headache and skin disorders (Table 4).

**Table 4:** Main adverse drug reactions in 553 patients treated with atovaquone-proguanil for imported uncomplicated malaria

**At Day 3 (N = 469 (%))**	**n (%)**
Digestive adverse reactions	115 (24.5)
*Nausea or vomiting*	*82 (17.5)*
*Diarrhoea*	*16 (3.4)*
*Abdominal pain*	*10 (2.1)*
*Others*	*7 (1.5)*
Cutaneous	10 (2.1)
Headache	18 (7.4)
Myalgia	3 (0.6)
Arthralgia	3 (0.6)
Anxiety	1 (0.2)
Total ADR° at Day 3	150 (32.0)
At Day 7 (N = 364 (%))*	
Digestive adverse reactions	11 (3.0)
*Nausea*	*3 (0.8)*
*Diarrhoea*	*4 (1.1)*
*Others*	*4 (1.1)*
Cutaneous	7 (1.9)
Headache	7 (1.9)
Total ADR° at Day 7	25 (6.9)
At Day 30 (N = 265 (%))*	
Digestive adverse reactions	1 (0.4)
*Nausea*	*0*
*Diarrhoea*	*1 (0.4)*
Cutaneous	1 (0.4)
Headache	0
Total ADR* at Day 30°	2(0.8)

*: Differences between denominators are due to patients lost to follow up.

ADR: Adverse drugs reactions.

In the multivariate analysis, when adjusted to origin, type of travel and parasitaemia at diagnosis, backpackers were more likely to experiment digestive ADR at day 3 than VFR (OR = 3.8 CI 95% [1.8-8.2]) (Table 5).

**Table 5:** Digestive adverse drug reactions under atovaquone-proguanil

		**Number**	**Digestive ADR day 3**		**Crude OR**		***p***	**Adjusted OR**		***p***
			**N**	**(%)**						
Sex	Male	306	74	(24.2)	1		*0.82*			
	Female	163	41	(25.1)	0.9	[0.6 - 1.4]				
Age	≤ 30	131	39	(29.8)	1		*0.36*			
	31-40	129	27	(20.9)	0.6	[0.3 - 1.1]				
	41-50	121	30	(24.8)	0,8	[0.4 - 1.3]				
	> 50	88	19	(21.6)	0.6	[0.3 - 1.2]				
Origin	African	415	92	(22.2)	1		*<0.01*	1		*0.19*
	European	43	16	(37.2)	2.1	[1.0 - 4.0]		1.2	[0.5 - 2.8]	
	Others	11	7	(63.6)	6.1	[1.7 - 21.4]		3.5	[0.9 - 14.0]	
Immunity	Non immune	233	60	(25.7)	1		*0.54*			
	Semi-immune	236	55	(23.3)	0.88 [06 – 1.3]					
Nausea at presentation	No	329	82	(24.9)	1		*0.76*			
	Yes	140	33	(23.6)	0.93 [0.58 – 1.48]					
Chronic illness	No	425	105	(24.7)	1		*0.77*			
	Yes	44	10	(22.7)	0,9	[0.4 - 1.9]				
Chemoprohylaxis	No	192	50	(26.0)	1		*0.51*			
	Yes	277	65	(23.5)	0.9	[0.6 - 1.3]				
Type of travel	VFR*	378	80	(21.2)	1		*< 0.01*	1		*< 0.01*
	Hostel	10	4	(40.0)	2.5	[0.7 - 9.0]		2.8	[0.8 - 10.4]	
	Backpackers	30	15	(50.0)	3.7	[1.7 - 7.9]		3.8	[1.8 - 8.2]	
	Unknown	51	16	(31.4)	1.7	[0.9 - 3.2]		1.7	[0.9 - 3.9]	
Parasitaemia at diagnosis	≤ 0.10%	144	27	(18.7)	1		*0.29*	1		*0.26*
	0.11 - 0.50%	148	42	(28.4)	1.7	[1.0 - 3.0]		1.8	[1.0 - 3.1]	
	0.51 - 1.00%	52	11	(21.2)	1.2	[0.5 - 2.5]		1.3	[0.6 - 2.9]	
	> 1%	90	25	(22.8)	1.7	[0.9 - 3.1]		1.9	[1.0 - 3.5]	
	Unknown	35	10	(28.6)	1.7	[0.7 - 4.0]		1.8	[0.7 - 4.2]	
Parasitaemia at day 3	Negative	290	72	(24.8)	1		*0.97*			
	Positive	128	31	(24.2)	0.9	[0.6 - 1.6]				
	Unknown	51	12	(23.5)	0.9	[0.5 - 1.9]				

*Visiting friends and relatives.

Assessment of tolerance by patients for the 437 who answered to the questionnaire was classified as good for 304 (69.6%), satisfactory for 116 (23.8%), bad for 41 (8.4%) and very bad for 27 (5.5%). In 20 cases, a switch to another drug was reported mainly because of vomiting (n = 15, 75%), confusion (n = 2, 10%), headache (n = 1, 5%), cutaneous eruption (n = 1, 5%), and suspected resistance because of a positive smear at day 3 (n = 1, 5%).

## Discussion

This ‘real life condition’ prospective, observational study of 553 patients treated by AP for uncomplicated *P. falciparum* malaria seems to be the largest series assessing the use of AP in the field of imported malaria. Patient profile (mostly young male adults of African origin living in Europe and infected in West Africa) are similar to those observed in the majority of studies on imported malaria [18-24,26-28]. The high percentage of HIV-infected patients (4.4%) observed in the study may be explained by the fact that the majority of patients are of African origin and because the nine recruiting centres are travel clinics linked to infectious diseases departments where a majority of the HIV patients living in Paris area are followed.

Not surprisingly, the rate of chemoprophylaxis and exposure prophylaxis was low. Clinical and biological presentation had no specificity but it is of interest to note that nearly one third of patients had no fever at admission, which might be misleading for non-experienced practitioners. Compared to some other studies describing non-comparative cohorts of malaria patients, follow-up, even too low, might be considered satisfactory given such a ‘real life’ design for this cohort, since outcome data are available for the majority at day 3 and since nearly half of patients were seen one month after treatment [16,19-21,26-29]. By contrast the loss to follow-up rate at one month in the recent European study was much lower, at 25% [4].

With regard to efficacy, if a majority of patients (95%) were fever-free at day 3, nearly a third of them were still parasitaemic confirming that AP is slow-acting [15,16,19,20,30,31]. The analysis of efficacy in a per-protocol approach gives a cure rate of 99% (three relapses of 265 patients with a follow-up at one month) which is comparable to other treatments [15,16,22,32,33]. Details on relapses were available for only two cases. In both cases the reason was probably suboptimal plasmatic AP level: consecutive to obesity (115 kg) in one case and to a poor absorption in the second case, since the patient had not taken food with the drug [34]. As a consequence, physicians should re-assess AP dosage in obese patients and should insist on food intake with AP to optimize its absorption.

Even though rare under AP and comparable to other malaria treatment (1% in this study’s series), the risk of relapse has to be considered by physicians given the potential severe outcome at a time when the diagnosis of malaria may be omitted (long delay after travel in endemic area) [4,21,23]. Given that relapses occur usually between day 14 and day 30 (and in a few cases later) after treatment, physicians should organize a ‘recapture’ system for patients lost to follow-up after day 7, even limited to a phone call, in order to identify promptly a possible recrudescence [4,35].

Perceived efficacy of AP was satisfying. This evaluation is not as valid as parasitological efficacy but it has not been studied in Africa before, both for curative treatment and for prophylaxis in travellers. It is considered as a significant predictor for compliance to treatment and preventative behaviours [36,37].

With regard to tolerance of AP, data of this study highlighted a high rate of digestive ADR, mainly at day 3, especially nausea and vomiting. Digestive ADR represented 77% of ADR reported at day 3 and 72% of the totality of ADR reported during follow-up. It seems that digestive ADR were more frequent in backpackers compared to migrants, with no particular explanation, and no comparable data were found in the literature. In 18% of cases (n = 15), vomiting was severe enough to justify a change to second-line treatment. This high occurrence of digestive ADR could be partially explained by the population enrolled in the study since black people are known to have a lower clearance rate of atovaquone compared to white people [38]. Yet there were no relationship between immunity, or African origin, with digestive ADR.

Surprisingly, these digestive side effects are one of the most important from studies. A review of ten trials comparing Atovaquone-Proguanil (AP) with other anti-malarial drugs for uncomplicated malaria report a median rate of nausea and/or vomiting (inter quartile range) of 15.6% (5.2 – 25.0) for Atovaquone-Proguanil whereas other studies did not report this ADR [10,11,15,16,18,20,30,31],[39]. To discriminate digestive ADR from symptoms related to malaria is difficult in a cohort and only clinical trials would be able to make the distinction. This study didn’t compare AP to other drugs, and this misclassification could be a bias. Nausea at diagnosis was not associated with digestive ADR at day 3, which involves AP rather than acute malaria in the etiology of these adverse effects.

Nevertheless, data from this series on digestive ADR are comparable to the results of a recent cohort study showing a higher switch rate to second-line treatment in patients treated with AP, compared to others [4].

The design of this study does not enable to guide physicians on their choice of anti-malarial drug. However, the significant risk of vomiting associated with AP needs to be taken into account, especially in patients already complaining of nausea and vomiting at the time of diagnosis. The need for fat intake with AP, as is recommended to improve absorption, may be an additional risk factor for vomiting [38]. Prescription of metoclopramide is probably not a solution since it decreases the bioavailability of atovaquone [40].

As observed in the literature, this study did not reveal any liver toxicity. Moderate variations in transaminase level observed at day 3 and 7 were not significant and were possibly due to malaria parasite itself [12,14,30,41].

A drop in haemoglobin level, as observed here, was commonly reported after initiation of treatment of acute malaria due to malaria haemolysis [42]. The absence of correlation between haemoglobin at diagnosis and initial parasitaemia is in line with other studies [43].

In 2007, French experts’ consensus recommended both AP and artemether-lumefantrine as first-line treatment for acute uncomplicated malaria [5]. As a consequence and because artemether-lumefantrine, even if registered throughout Europe in 1999, is only available since 2007 in France, the use of AP has progressively increased from 25% in 2006 to 46% in 2011 [3]. On the one hand, AP’s good efficacy was confirmed by this series while its limitations were described (mainly slow-acting drug, poor absorption, and ADR, such as vomiting). On the other hand, use of artemether-lumefantrine in the particular situation of imported malaria still has a limited experience. Hence, regarding imported malaria, the question of which association between AP or artemisinin combination therapy (ACT) is the best option remains. A clinical trial is currently comparing the use of AP and ACT in the indication of uncomplicated malaria in non-endemic areas and will hopefully bring the answer [44].

With the introduction of ACT in Europe (artemether-lumefantrine and, since mid-2012, dihydroartemisinin-piperaquine are both authorized in France and in a limited number of European countries) for uncomplicated acute malaria onset, the use of AP will probably decrease in favour of ACT, due to its prompt efficacy and good tolerance.

## Conclusion

This observational series of 553 cases, the largest to date in number of patients, describes a large experience in using AP for imported uncomplicated malaria in real life conditions. Despite a non-comparative design, it appears that its efficacy is good and comparable to other similar drugs. The study confirms that AP is a valuable treatment option, while ADR, such as vomiting and its limited absorption in some cases, may be a limitation for its use.

## Competing interests

The authors have declared that they have no competing interests.

## Authors’ contributions

HC, PR and OB participated in the design of the study and performed the statistical analysis. PR and OB conceived of the study, and participated in its coordination. HC, JC, SM and OB contributed to the draft manuscript. OF, PB, PC, HG, PHC, NG and MB participated in the recruitment of included patients. All authors read and approved the final manuscript.
